# Deptor transcriptionally regulates endoplasmic reticulum homeostasis in multiple myeloma cells

**DOI:** 10.18632/oncotarget.12060

**Published:** 2016-09-16

**Authors:** Valeria Catena, Tiziana Bruno, Francesca De Nicola, Frauke Goeman, Matteo Pallocca, Simona Iezzi, Cristina Sorino, Giovanni Cigliana, Aristide Floridi, Giovanni Blandino, Maurizio Fanciulli

**Affiliations:** ^1^ SAFU, Department of Research, Advanced Diagnostics, and Technological Innovation, Translational Research Area, Regina Elena National Cancer Institute, 00144, Rome, Italy; ^2^ Epigenetic, Department of Research, Advanced Diagnostics, and Technological Innovation, Translational Research Area, Regina Elena National Cancer Institute, 00144, Rome, Italy; ^3^ Clinical Pathology Laboratories, Department of Research, Advanced Diagnostics, and Technological Innovation, Translational Research Area, Regina Elena National Cancer Institute, 00144, Rome, Italy

**Keywords:** multiple myeloma, deptor, ER stress, homeostasis, transcription

## Abstract

Multiple myeloma (MM) is a malignant disorder of plasma cells characterized by active production and secretion of monoclonal immunoglobulins (IgG), thus rendering cells prone to endoplasmic reticulum (ER) stress. For this reason, MM cell survival requires to maintain ER homeostasis at basal levels. Deptor is an mTOR binding protein, belonging to the mTORC1 and mTORC2 complexes. It was reported that Deptor is overexpressed in MM cells where it inhibits mTOR kinase activity and promotes cell survival by activating Akt signaling. Here we identify Deptor as a nuclear protein, able to bind DNA and regulate transcription in MM cells. In particular, we found that Deptor plays an important role in the maintenance of the ER network, sustaining the expression of several genes involved in this pathway. In agreement with this, Deptor depletion induces ER stress and synergizes the effect of the proteasome inhibitor bortezomib (Bz) in MM cells. These findings provide important new insights in the ER stress control in MM cells.

## INTRODUCTION

Multiple myeloma (MM) is a malignant pathology characterized by the proliferation of clonal plasma cells (PC) in the bone marrow [1]. It is the second most frequent haematological malignancy in the USA and Europe [2] with a median age at diagnosis of 69 years [3]. Despite several improvements in supportive cure, MM remains incurable and patients invariably relapse [4]. For this reason, many efforts have focused on identifying novel strategies in eradicating the subset of tumor cells that escape from therapy. One of the typical features of MM cells is represented by the massive production and secretion of immunoglobulins (Igs)[5], often detectable in patient serum and/or urine, and the ability to adapt to prolonged endoplasmic reticulum (ER) stress [6]. Indeed, MM cells are able to reduce ER protein load by increasing degradation of the accumulated proteins through the activation of an adaptive response called Unfolded Protein Response (UPR) [7]. The UPR, in MM cells, is regulated by the ER chaperone BiP (Binding immunoglobulin Protein),[8] which coordinates events leading to an attenuate general protein translation and rapid degradation of exceeding proteins [9, 10]. However, extensive ER stress induces activation of the pro-apoptotic UPR effector CHOP (transcription factor C/EBP Homologous Protein), involved in downregulating Bcl-2 expression and in activating BAX [11]. Currently, MM cells have demonstrated their sensitivity to compounds that target protein homeostasis, such as proteasome inhibitors (PIs) [12]. In particular, PIs block ER-Associated Degradation (ERAD) mechanisms, leading to accumulation of unfolded proteins and induction of ER stress and apoptosis. Therefore, therapeutic strategies targeting the ER stress response pathways may be considered promising to improve clinical outcome of MM patients.

Deptor, also named DEPDC6 (DEP- Domain-Containing Protein-6) is a 48 kDa protein, playing a critical role in mTOR pathway [13]. It is a component of mTORC1 and mTORC2 complexes and specifically interacts with mTOR, inhibiting thereby its kinase activity [13]. Notably, the presence of a negative feed-forward loop in which Deptor and mTOR regulate each other was demonstrated [14, 15]. Indeed, Deptor downregulation leads to an increase in mTOR activity, which in turn produces reduction of Deptor expression [13]. In several tumors Deptor has been found to be overexpressed, playing an important role in many cellular processes, such as cell growth, apoptosis, autophagy and drug resistance [16]. Importantly, in MM cells high levels of Deptor lead to an enhanced Akt kinase activity, thus providing a pro-survival effect [13]. Consistent with this notion, Deptor expression is correlated with poor survival in MM patients [17]. Moreover, it was hypothesized that Deptor may affect the protein synthesis machinery by inhibiting the mTORC1 activity, thereby protecting MM cells against ER stress and inhibiting apoptosis [18]. Nevertheless, the complete role played by Deptor in this pathology remains elusive.

In this study, we found Deptor as a nuclear protein, accumulated in the chromatin fraction. Strikingly, we produced evidence showing that Deptor regulates the transcription of several genes involved in the control of ER homeostasis and that its downregulation increases ER stress, thus inducing activation of apoptotic response. Finally, our results highlight how the depletion of Deptor makes MM cells more sensitive to the treatment with the proteasome inhibitor bortezomib (Bz). In aggregate, our results suggest Deptor as a novel target for MM treatment.

## RESULTS

### Deptor is a nuclear protein

**Figure 1:**
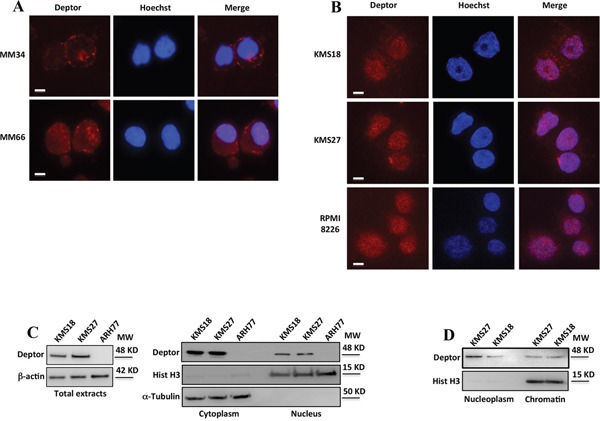
Deptor is a nuclear protein **A-B.** Immunofluorescence analysis of Deptor expression in plasma cells purified from primary myelomas (A) and in MM cell lines (B). Cells were fixed in 4% formaldehyde for 15 min and then permeabilized with 0.1% Triton X-100. Nuclei were visualized by staining with Hoechst dye. Scale bar represents 10 μm; 63X original magnification. **C.** Western blot (WB) analysis, with the indicated antibodies (Abs), of total (left), nuclear and cytoplasmic extracts (right) from KMS27, KMS18 and ARH77 MM cell lines. **D.** WB analysis of soluble nuclear and chromatin-bound protein fractions with the indicated Abs from KMS27 and KMS18 MM cell lines.

Although it has been demonstrated that Deptor is a potent mTOR inhibitor,[13] a complete characterization of its functions has still not been well elucidated. In contrast with the most cancer types in which its expression is generally low, Deptor exhibits high levels in MM,[16] leading to presume an important role of this protein in this pathology. To shed light on Deptor functions within MM cells, we started investigating its localization within these cells. Therefore, we analyzed Deptor expression by immunofluorescence in human CD138^+^ primary myelomas (MM34, MM66)[16] observing a clear nuclear signal of Deptor (Figure 1A). These results were confirmed in several MM cell lines (KMS27, KMS18 and RPMI 8226) (Figure 1B). To confirm the presence of Deptor in the nuclear compartment, we performed subcellular fractionation in KMS18, KMS27 and ARH77, cells with different levels of Deptor. As shown in Figure 1C, in addition to a cytoplasmic signal, a nuclear anti-Deptor positive staining was also detected. To further characterize this localization, chromatin-bound proteins were extracted from KMS18 and KMS27 cells displaying a significant accumulation of Deptor (Figure 1D).

Altogether, these results demonstrate a nuclear localization of Deptor, leaving room to assume its additional functions.

### Deptor modulates transcription of genes involved in ER homeostasis

**Figure 2:**
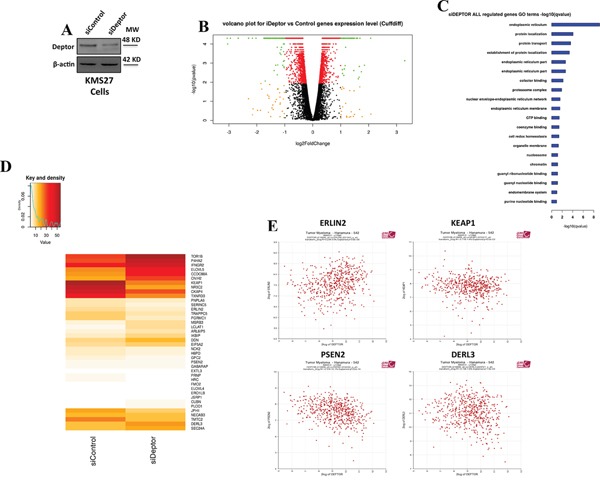
Deptor modulates transcription of genes involved in ER homeostasis **A.** WB analysis with the indicated Abs of total cell extracts (TCEs) from KMS27 cells transiently transfected with Stealth siRNA negative control (siControl) or siRNA Deptor (siDeptor). **B.** Volcano Plot of all assembled RNA-seq genes performed with KMS27 cells transiently transfected as in A. The image depicts genes with strong modulation but not significant (qvalue < 0.05) in orange; significant modulation but mild fold-change (abs (log2fc) < 1) in red; significant and strongly modulated genes in green. **C.** Gene Ontology significance barplot of all siDeptor modulated genes deriving from Cuffdiff analysis. **D.** Intensity (FPKM) heatmap of endoplasmic reticulum genes (top) and ER stress genes (bottom) modulated after Deptor silencing. **E.** Correlation analysis of expression values from 550 myeloma patients, as described in Zhan et al [28], between Deptor and endoplasmic reticulum genes (ERLIN2, KEAP1, PSEN2 and DERL3) (probe set 218858_at for DEPTOR). R2 values: Erlin2=0,234; KEAP1=−0,118; PSEN2=−0,318; DERL3=−0,136.

The above results show that Deptor is present in the chromatin fraction. Based on this indication, we wondered whether this protein was somehow involved in transcriptional regulation. To verify this hypothesis, we performed a RNA-seq analysis by using mRNA from KMS27 cells transfected with siRNA Deptor or siRNA negative control (Figure 2A). Differential expression analysis of this experiment revealed 1891 transcripts significantly regulated (968 down, 923 up) in response to Deptor depletion (Figure 2B), thus confirming that Deptor is able to regulate transcription.

To identify the cellular pathways affected by Deptor depletion, we performed a Gene Ontology analysis of differentially expressed genes. From this analysis, we observed that Deptor expression lead to sustaining of several pathways involved in protein transport or localization (Figure 2C and 2D). Notably, among them, we found that Deptor exhibits a strong change of expression levels of many genes involved in endoplasmic reticulum (ER) homeostasis, a crucial event in maintaining MM cell survival (Figure 2C and 2D).

In order to confirm these findings, we conducted a correlation analysis of microarray public data of 550 MM patients [28]. Interestingly, we found that several ER homeostasis genes, such as *ERLIN2, KEAP1, PSEN2* and *DERL3* exhibit a significant correlation with Deptor mRNA expression (Figure 2E) [29]. Furthermore, we performed a Gene Ontology enrichment over all the transcripts significantly correlated with Deptor (Minus Set: 2033 probes, Plus Set: 2144, p value < 0.01) in the Hanamura MM Dataset of R2 [28]. Several significant clusters support of hypothesis of Deptor role in MM, such as endoplasmic reticulum and transcription initiation from RNA polymerase II promoter (Supplementary Table S1).

**Figure 3:**
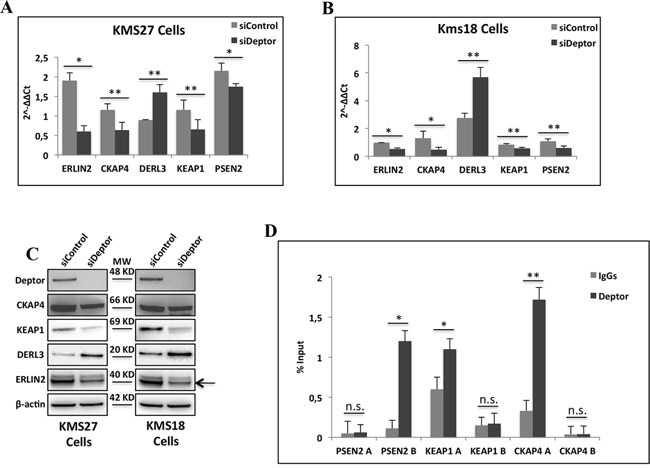
Deptor modulates transcription of genes involved in ER homeostasis **A-B.** Quantitative RT–PCR (qRT–PCR) for ER homeostasis gene expression was performed in KMS27 (A) and KMS18 (B) cells transiently transfected with Stealth siRNA negative control (siControl) or siRNA Deptor (siDeptor). Values were normalized to RPL19 mRNA expression. Error bars represent the standard error of three different experiments. *P = 0.002, **P≤0.03 (A); *P≤0.0002, **P≤0.01 (B). **C.** WB analysis with the indicated Abs of TCEs from KMS27 and KMS18 cells transfected as in A and B. Arrowhead indicates specific ERLIN2 protein band. **D.** ChIP-qPCR analysis of KMS27 cells using anti-Deptor Ab or control IgGs. Primer were designed to amplified two different promoter regions of CKAP4, ERLIN2 and PSEN2. Error bars represent the standard error of three different experiments. n.s., not significant, *P≤0.0004, **P=0.0225.

These data were further validated by quantitative real-time PCR (qRT–PCR) analysis of mRNA from KMS18 and KMS27 cells transfected with siRNA Deptor or siRNA negative control (Figure 3A and 3B). Consistent with these results, western blot analysis from these cells revealed that Deptor depletion produced a significant reduction of ERLIN2, KEAP1, PSEN2 protein levels, with a concomitant increase of DERL3 amounts (Figure 3C) [30–32]. In agreement, ectopic over-expression of Deptor in U266 cells, a MM cell line with low expression of this protein, produced an increase of ERLIN2, KEAP1 and CKAP4 protein levels with a concomitant decrease of DERL3 expression (Supplementary Figure S1A).

To verify that the regulation of transcription observed above was a direct effect of Deptor and not via a regulation of the mTORC1 activity, we carried out a quantitative ChIP-qPCR assay in KMS27 cells. This experiment showed the presence of Deptor on specific promoter regions of *PSEN2*, *CKAP4* and *KEAP1* genes (Figure 3D), confirming the direct involvement of Deptor in gene transcription.

### Deptor depletion enhances ER stress in MM cells

**Figure 4:**
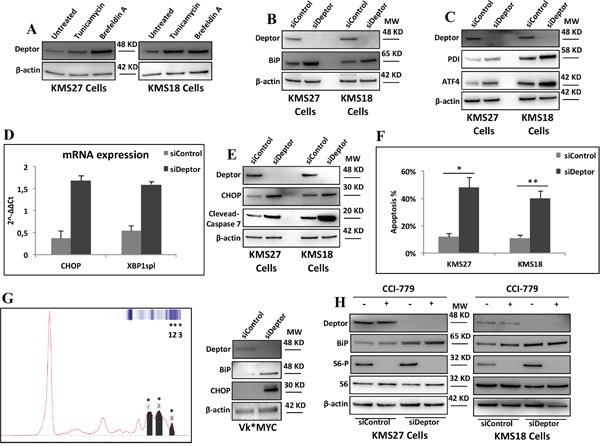
Deptor depletion enhances ER stress in MM cells **A.** WB analysis with the indicated Abs of TCEs from KMS27 and KMS18 cells treated where indicated with tunicamycin (2mg/ml) or brefeldin A (1μg/ml) for 8hrs. **B.** WB analysis with the indicated Abs of TCEs from KMS18 and KMS27 cells transiently transfected with Stealth siRNA negative control (siControl) or siRNA Deptor (siDeptor). **C.** KMS18 and KMS27 cells, transfected as in B, were analyzed by WB with the indicated Abs. **D.** qRT-PCR for XBP1spl and CHOP mRNA expression was performed after transient transfection of KMS27 cells as in B. Values were normalized to RPL19 mRNA expression. Error bars represent the standard error of three different experiments performed in duplicate. P≤0.0001. **E.** KMS27 and KMS18 cells were transfected as in B and then analyzed by WB with the indicated Abs. **F.** KMS18 and KMS27 cells were transfected as in B and after 60 hrs assayed for cell death by trypan blue staining and percentages represent trypan blue-incorporating cells. Data are presented as the mean SD from three independent experiments performed in duplicate. *P=0.0007, **P=0.0433. **G.** Left: representative Serum Protein Electrophoresis (SPEP) performed on a Vk*MYC mouse. Asterisks emphasize M-spikes, hallmark of MM disease, detected in mouse serum. Right: WB analysis with the indicated Abs of TCEs of CD138^+^ neoplastic cells from Vk*MYC mice transiently transfected as in B. **H.** KMS27 cells were transfected as in A and, where indicated, treated with 100nM CCI-779 for 16 hrs and then analyzed by WB using the indicated Abs.

Several studies demonstrated that MM cells actively produce and secrete a massive amount of immunoglobulins (Igs) responsible for ER stress in these cells [5, 6]. For this reason, MM cells react with an adaptive response to ER stress, termed Unfolded Protein Response (UPR) [7]. On the basis of the results shown above, we speculated whether Deptor might play an important role in keeping ER stress under control in MM cells. As shown in Figure 4A, Deptor levels raised in response to ER stress induced by treating MM cells with tunicamycin or brefeldin A [33]. Next, we evaluated the effects of Deptor inhibition on ER stress. As shown in Figure 4B, Deptor depletion induced a strong rise in BiP levels, a master regulator of the UPR [8, 34], in both KMS18 and KMS27 cells, indicating UPR induction. Once UPR is induced, BiP dissociates from three important sensors, PERK, ATF6 and IRE1α, activating them accordingly [30, 35–36]. This event triggers a signaling cascade, leading to the activation of several downstream targets, such as ATF4, or XBP1 splicing (XBP1spl) [33]. To confirm that Deptor inhibition is responsible for increased UPR signaling, we carried out experiments depleting Deptor in KMS18 and KMS27 MM cell lines and observed that Deptor inhibition activated PERK and IRE1α signaling, as highlighted by the increase in protein levels of ATF4 and XBP1 mRNA splicing, respectively (Figure 4C and 4D). Consistent with these results, Deptor depletion produced an up-regulation of PDI, a well-known target of XBP1spl (Figure 4C).

Since MM cells are exceptionally sensitive to apoptosis induced by ER stress,[6] we investigated whether Deptor depletion was able to increase apoptosis. It is for this purpose we measured the induction of CHOP, an effector of ER stress induced apoptosis, in MM cells depleted, or not, for Deptor expression. Both mRNA and protein levels of CHOP were increased after silencing of Deptor (Figure 4D and 4E). Consistent with these findings, Deptor depletion led to elevated apoptosis rate in KMS27 and KMS18 cells (Figure 4F).

To further confirm these results, we isolated CD138+ MM cells from Vk*Myc mice [22] exhibiting advanced MM disease (Figure 4G), and transfected them with siDeptor or siControl oligos. As shown in Figure 4G, western blot analysis of TCEs from these mice confirmed that Deptor depletion induces an increase in BiP and CHOP protein levels, indicating ER stress.

Previously, it has been demonstrated that overexpression of Deptor inhibits mTORC1 activities promoting MM survival [13]. Based on this evidence, we wondered whether Deptor depletion induced apoptosis by activating mTORC1, subsequently leading to Akt inhibition. To assess the relevance of this mechanism, we treated Deptor-depleted and control MM cells with the mTORC1 inhibitor, CCI-779. As shown in Figure 4H, CCI-779 treatment did not have a significant effect on BiP increase induced by Deptor depletion, thus indicating that Deptor activity on mTORC1 is not required for maintaining ER homeostasis.

All together, these results indicate that Deptor expression is required for maintaining ER homeostasis in MM cells.

### Deptor sensitizes MM cells to bortezomib treatment

**Figure 5:**
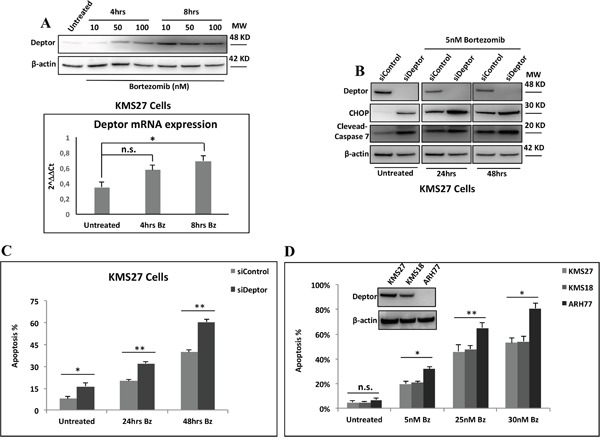
Deptor sensitizes MM cells to Bz treatment **A.** Top: WB analysis with the indicated Abs of TCEs from KMS27 cells treated or not with different concentrations of Bz for 4hrs and 8hrs. Bottom: qRT-PCR for Deptor mRNA expression of KMS27 cells treated as in A. Values were normalized to RPL19 mRNA expression. Error bars represent the standard error of three different experiments performed in duplicate. n.s., not significant, *P<0.03. **B.** WB analysis with the indicated Abs of TCEs from KMS27 cells transfected with Stealth siRNA negative control (siControl) or siRNA Deptor (siDeptor) and after 24hrs treated or not with 5nM Bz for further 24 and 48hrs. **C.** Cell death detection of KMS27 cells treated like in B and assayed by trypan blue staining. Percentages represent trypan blue-incorporating cells. Data are presented as the mean SD from three independent experiments performed in duplicate. *P=0.0433, **P≤0.01. **D.** MM cells overexpressing (KMS27 and KMS18) or lacking (ARH77) of Deptor expression were treated with the indicated concentrations of Bz for 48hrs and cell death was assayed by trypan blue staining. Percentages represent trypan blue-incorporating cells. Data are presented as the mean SD from three independent experiments performed in duplicate. n.s., not significant, *P≤0.01, **P≤0.03.

The overall treatment of MM patients has been significantly improved by proteasome inhibitors (PIs),[12] which activate cell apoptosis through different mechanisms [37]. Currently, the single most important class of anti-myeloma therapeutics is the PI, such as bortezomib (Bz). Many studies have demonstrated that Bz treatment rapidly activates PERK, which, in turn, phosphorylates eIF2α. This event leads to a general decrease of *de novo* protein synthesis and an increase in ATF4 translation, followed by CHOP expression which drives the cells into apoptosis [6]. In agreement with these observations, we observed an increase of Deptor levels in response to Bz treatment in MM cell lines (Figure 5A). Therefore, to verify whether Deptor silencing was able to enhance the pro-apoptotic effect of this drug, we treated KMS27 cells depleted or not for the Deptor expression with Bz at different times. As shown in Figure 5B, Bz led to an increase of CHOP expression which was further enhanced upon Deptor depletion. Accordingly, Deptor depletion increased also the Bz-induced apoptosis (Figure 5C). To further confirm these results, we treated various MM cell lines expressing different levels of Deptor with Bz. As shown in Figure 5D MM cells with lower levels of Deptor expression showed greater sensitivity to Bz treatments. Consistent with these findings, U266 cells over-expressing Deptor exhibited a strong reduction of apoptosis induced by Bz, indicating that Deptor expression can contribute to protect MM cells against cell death (Supplementary Figure S1B).

Taken together these data suggest that MM cells can be sensitized to PIs in combination with Deptor silencing and thereby enhancing the expression of terminal UPR components.

## DISCUSSION

Deptor is an important factor involved in controlling mTOR activity. Indeed, this protein is a negative regulator of the mTORC1 and mTORC2 signaling pathways by inhibiting the kinase activity of both complexes [13]. High levels of Deptor, in MM cells, sustain mTORC2 and Akt activity,[13] thereby promoting cell growth, survival, adipogenesis and metabolic switch [13, 38, 39].

In this study we provide evidence of a novel role for Deptor as a regulator of ER homeostasis in MM cells. Indeed, here we describe Deptor as a nuclear protein and it is found to bind DNA and regulate transcription. In particular, we show that Deptor modulates the expression of several genes, regulating important pathways in MM cells such as ER homeostasis, protein transport and proteasome complex. Consistent with these results, in these cells Deptor depletion increases ER stress response and induces apoptosis. In addition, we demonstrate that Deptor protein levels correlate with Bz resistance in MM cells and vice versa that inhibition of Deptor expression sensitizes these cells to this drug.

A relevant finding of this study is the identification of the nuclear localization of Deptor (Figure 1). This feature was observed in MM patients-primary cells as well as in cell lines, and a significant amount of Deptor was found to be associated with chromatin. Interestingly, nuclear Deptor was also observed in several other cell lines, such as human HCT116, HeLa and human embryonic kidney (HEK) 293 cells (data not shown). A nuclear localization of mTOR was extensively described [40, 41]. In particular, this kinase was found to interact with TFIIIC and to be recruited, together with Raptor, to tRNA and 5S rRNA genes [42–44]. Therefore, it is conceivable that Deptor could be recruited into the nucleus through its direct interaction with mTOR. However, our findings indicate that Deptor is able to regulate mRNA transcription, suggesting a new role for this protein in addition to its ability to inhibit mTOR. Further experiments will better characterize the mechanisms by which Deptor is present in the nucleus.

An RNA-seq analysis showed that several genes are significantly modulated by Deptor depletion in KMS27 cells (Figure 2). These findings identify this protein as an important regulator of transcription. Moreover, ChIP experiments confirmed the presence of Deptor onto the promoter of several genes modulated by its inhibition (Figure 3D). At this time it is not clear whether Deptor has the capacity to directly bind the DNA or if its activity is mediated by an interaction with some factors of the transcriptional apparatus like it has already been shown for mTOR. The Gene Onthology analysis of differentially expressed genes revealed that Deptor regulates several pathways involved in protein processing and in their localization (Figure 2C). In particular, Deptor was found to exert an important role in ER homeostasis, regulating many genes involved in this pathway, such as ERLIN2, PSEN2 and KEAP1 (Figure 2D). In agreement with these findings, we revealed a significant expression correlation between Deptor and several of its targets analyzing a database with 559 MM patients (Figure 2E) [28]. The relevance of the role played by Deptor in ER homeostasis was also confirmed by the evidence that Deptor depletion up-regulates DERLIN-3, a transmembrane protein involved in UPR-induced ERAD (ER associated degradation) by forming a channel that allows the retrotrascription of un-/misfolded proteins from the ER lumen to the cytosol [31]. Moreover, Deptor inhibition induced the release of BiP from ER stress sensors, such as PERK and IRE1α, leading to increased expression of ATF4, CHOP and XBP1spl, and to apoptosis activation (Figure 4).

MM cells are characterized by a high demand on the protein synthesis machinery to produce and secrete huge amounts of immunoglobulins. This phenomenon inevitably results in high levels of ER stress rendering these cells more susceptible to apoptosis [5, 6]. Therefore, in order to survive, MM cells need to reduce the loading on the ER by activating UPR signaling and by inducing genes involved in ER homeostasis [10]. Here, we demonstrate that Deptor, which is highly overexpressed in MM, is a crucial regulator of homeostasis, inhibiting thereby ER stress induced apoptosis. A tissue-specific RNA expression pattern analysis revealed high levels of Deptor in skeletal muscle, thyroid, adrenal and salivary glands,[45] all tissues where the endoplasmic reticulum plays a prominent role in the regulation of both muscle contraction and protein secretion, thus reinforcing the notion that Deptor is required for the regulation of ER functions.

It has been demonstrated that ER stress is often coupled with mTORC1 activation, suppression of Akt activity and induction of apoptosis [46]. On the basis of these observations, it is therefore possible to support the hypothesis of a dual coordinate function of Deptor adapted to control mTOR activity in MM cells.

Autophagy is another important pathway that protects MM cells against ER stress [47]. Previous studies have reported that Deptor induces autophagy by suppressing mTOR activity in response to stress [48, 49] and, in the absence of Deptor, a decrease of the autophagy pathway occurs, leading to a greater MM cell death rate [19]. Also in this case, the control of the endoplasmic reticulum homeostasis by Deptor can contribute in maintaining the levels of autophagy and therefore inhibit the activation of apoptosis.

Bz, one of the major drugs used for the treatment of MM, is a proteasome inhibitor that is able to activate several components of UPR [6, 50]. In agreement with previous results,[48] we observed that MM cell lines, expressing low levels of Deptor, exhibited more sensitivity to Bz than those bearing high Deptor expression (Figure 5D). Consistent with these data, Deptor depletion significantly increases the sensitivity of KMS27 and KMS18 cells to Bz, unlike its over-expression in U266 cells protects against Bz activity (Figures 5A, 5B and S1B). Together, these findings identify a new role for Deptor in MM survival and suggest Deptor as a new molecular target to improve the outcome of therapy preventing relapse and resistance in MM.

## MATERIALS AND METHODS

### Multiple myeloma-derived cells, transfection and reagents

Multiple myeloma (MM) patient samples were collected as part of routine clinical examination and plasma cells were enriched as described in Desantis et al [19].

KMS18, KMS27, RPMI8226, U266 and ARH77 human MM cell lines were grown as previously described [19]. Transfections were carried out by Lipofectamine 3000 (Life Technologies) following the manufacturer's instructions. The plasmid pRK5 human Flag-Deptor was purchased from Addgene (ID 21334). The reagents used were: the mTOR inhibitor CCI-779 (Chemocare); Doxycycline (Sigma); the proteasome inhibitor Bortezomib/Velcade (Janssen); tunicamycin and brefeldin A (Sigma).

### Total cell extracts, nuclear extracts and chromatin isolation

Whole cell extracts were prepared as previously described [20]. The cytoplasmic and nuclear extracts were obtained from KMS18, KMS27, and ARH77 cells as described in Sorino et al [21].

Chromatin isolation was performed by resuspending cells in buffer A (10 mM HEPES pH 7.9, 10 mM KCl, 1.5 mM MgCl_2_, 0.34 M sucrose, 10% glycerol). NP-40 (0,05%) was added and the cells were incubated for 2 min on ice. Nuclei were collected by low-speed centrifugation for 5 min at 4°C and washed once in buffer A and then lysed in buffer B (3 mM EDTA, 0.2 mM EGTA, 1 mM DTT). Insoluble chromatin was collected by centrifugation for 4 min at 4°C and washed once in buffer B. The final chromatin pellet was resuspended in chromatin extraction buffer (500mM NaCl, 50mM Tris-HCl pH 7.5; 1.5 mM MgCl_2_; 0,5% Nonidet P-40) and sonicated for 20 sec, to release chromatin-bound proteins.

### Antibodies and western blot analysis

The rabbit polyclonal antibodies used were: Deptor (Upstate/Millipore, 09-463) I.F. 1:100; ERLIN2 (Sigma) WB 1:100; BiP (Cell Signaling, clone C50B12, #9956) WB 1:1000; PDI (Cell Signaling, clone C81H6, #9956) WB 1:1000; DERL3 (GeneTex, N3C2) WB 1: 1000; CKAP4 (GeneTex, N3C3) WB 1:1000; KEAP1 (Santa Cruz Biotechnology, clone E-20, sc-15246) WB 1:500; S6 p-S235/236 (Cell Signaling, #4858, clone D57.2.2E) WB 1:1000; Histone H3 (Abcam, clone ab1791) WB 1:1000; Cleaved caspase 7 (Cell Signaling, #9491) WB 1:1000; ATF4 (Santa Cruz Biotechnology, clone C-20, sc-200) WB 1:100. Mouse monoclonal antibodies: Deptor (Santa Cruz Biotechnology, clone A-3, sc-398169) WB 1:1000; β-actin (Sigma, clone AC-15, A5441) WB 1:10000; CHOP/GADD153 (Proteintech, clone 4D5A9) WB 1:500; S6 (Cell Signaling, clone 54D2) WB 1:1000; α-Tubulin (Calbiochem, clone DM1A) WB 1:1000; Flag (Sigma, clone M2, F1804) WB 1:1000. Secondary antibodies used were goat anti-mouse and goat anti-rabbit, conjugated to horseradish peroxidase (Biorad). Immunostained bands were detected by the chemiluminescent method (Pierce) and the images were acquired using Alliance system by UVITECH, Cambridge.

### Vk*MYC mice

Bone marrow samples were collected from Vk*MYC mice (kindly provided by M. Chesi, Mayo Clinic Arizona)[22] and enriched for plasma cells by magnetic cell separation using a mouse CD138 positive selection kit (Miltenyi-Biotec-Germany) and Macs Separator (Macs Miltenyi-Biotec-Germany). All procedures involving animals and their care were conducted in accordance with institutional guidelines and regulations [23].

### Cell death detection

KMS27, KMS18 and ARH77 cells were plated on 35 mm plates and treated with different concentrations of bortezomib. After 24 and 48 hrs, cell death was assayed by trypan blue staining. The percentages represent trypan blue-incorporating cells. Data are presented as the mean ±SD from three independent experiments performed in duplicate.

### Immunofluorescence

Immunofluorescence analysis with Deptor antibody in MM cell lines and in MM patient samples was performed as described in Desantis et al [19]. Primary antibody was used for immunostaining, followed by Alexa-Fluor-594-conjugated and Alexa-Fluor-488-conjugated anti-rabbit IgG (Life Technologies). Nuclei were visualized by staining with 1 μg/ml Hoechst dye 33258 (Sigma). Immunofluorescence images were acquired by using an Axioskop 2 plus microscope (Zeiss) equipped with 63X oil immersion objective and fluorescence signals were analyzed by using a CCD camera (Zeiss, Oberkochen, Germany).

### Chromatin immunoprecipitation assays (ChIP)

Chromatin immunoprecipitation assays in KMS27 cells were performed as previously described [24] by using an anti-Deptor antibody. Immunoprecipitations with no specific immunoglobulins (Santa Cruz) were performed as negative control. For quantitative ChIP analysis (ChIP-qPCR) 1μl of purified DNA was used for amplification on an Applied Biosystems 7500 Fast Real Time PCR system (Applied Biosystem SYBR GREEN).

The following promoter-specific primers were employed in PCR amplifications:

**Table:** 

CKAP4 A forward	5′-CAACACAACCCGAACTCAGC-3′
CKAP4 A reverse	5′-CTTTGGGGGCAGAAAGACCT-3′
CKAP4 B forward	5′-CAAGGTCTTTCTGCCCCCAAAG-3′
CKAP4 B reverse	5′-AAGCAACTTGCCCACTCACT-3′
PSEN2 A forward	5′-CCAAATCCTGGAACCACA-3′
PSEN2 A reverse	5′-GTCGGAACTAAGCGACGACC-3′
PSEN2 B forward	5′-CTCAACCTACCCCACAGACC-3′
PSEN2 B reverse	5′-GGCGGTAGGACATAGGCTTCG-3′
KEAP1 A forward	5′-AATTTTCCCTAGATCCTGCGGC-3′
KEAP1 A reverse	5′-GAAAGGAGCGGCGATTCTC-3′
KEAP1 B forward	5′-GATCGCTTGAGGCCAGGAGC-3′
KEAP1 B reverse	5′- GAAAGGAGCGGCGATTCTC-3′

### siRNA

Experiments of Deptor siRNA-mediated interference were performed by transfecting a specific pool of double-stranded RNA oligonucleotides (siDeptor) (Stealth, Life Technologies) using Lipofectamine 3000 (Life Technologies). Stealth siRNA negative control oligos (siControl) were purchased from Life Technologies.

### RNA isolation and quantitative RT-PCR analysis

KMS27 and KMS18 cell lines were harvested 60 hrs after transfection with siDeptor or siRNA negative control and total RNA isolated as described in Desantis et al [19]. The first-strand cDNA was synthesized with random primers and M-MLV reverse transcriptase (Life Technologies). The cDNA was used for quantitative real-time PCR (qRT-PCR) experiments as previously described [25]. ΔΔCt values were normalized with those obtained from the amplification of the endogenous RPL19 gene. Data are presented as the mean ±SD from three independent experiments performed in duplicate.

The following human specific primers were employed in PCR amplifications:

**Table:** 

CHOP forward	5′-CTGCCTTTCACCTTGGAGAC-3′
CHOP reverse	5′-CGTTTCCTGGGGATGAGATA-3′
XBP1S forward	5′-GAGTCCGCAGCAGGTG-3′
XBP1S reverse	5′-GTGTCAGAGTCCATGGGA-3′
KEAP1 forward	5′-CGTGGCTGTCCTCAATCGT-3′
KEAP1 reverse	5′-CCCCCAGCAGCATAGATACAGT-3′
ERLIN2 forward	5′-TTCAAGCTGTGCGGGTAACA-3′
ERLIN2 reverse	5′-CCACCTGGGCCACTTTTTCT-3′
CKAP4 forward	5′-AGTGAGGTCAGCCGGATCAG-3′
CKAP4 reverse	5′-GATGGCGATGTTGTCGTTGA-3′
PSEN2 forward	5′-CTGCCCAGGAGAGAAATGAG-3′
PSEN2 reverse	5′-CAGTCAAGGGAGGCTCAAAG-3′
DERL3 forward	5′-TGGAGCCCTGTCCAG-3′
DERL3 reverse	5′-GCCGAAGAAGTTGAC-3′
RPL19 forward	5′-CGGAAGGGCAGGCACAT-3′
RPL19 reverse	5′-GGCGCAAAATCCTCATTCTC-3′

### RNA-sequencing

After transfection with siDeptor or siRNA negative control using Lipofectamine 3000 (Life Technologies), total RNA was extracted from KMS27 cells using Qiazol (Invitrogen), purified from genomic DNA contamination through a DNase I (Qiagen) digestion step and further enriched by Qiagen RNeasy columns for gene expression profiling (Qiagen). Quantity and integrity of the extracted RNA were assessed by NanoDrop Spectrophotometer (NanoDrop Technologies) and by Agilent 2100 Bioanalyzer (Agilent Technologies), respectively. RNA libraries for sequencing were generated in triplicate according to the Illumina TruSeq Stranded Total RNA kit with an initial ribosomal depletion step using Ribo Zero Gold (Illumina, Inc., San Diego, CA, US). Starting material was 500 ng of total RNA. The libraries were quantified by qPCR and sequenced in paired-end mode (2x75 bp) with the Genome Analyzer IIx (Illumina, Inc., San Diego, CA, US). For each sample generated by the Illumina platform, a pre-process step for quality control was performed to assess sequence data quality and to discard low quality reads.

### Bioinformatic analysis

The analysis were performed exploiting the RNA-seq analysis workflow RAP,[26] that comprises read mapping, transcript assembly and abundancy estimation followed by transcript-based differential expression via the Tuxedo suite [27]. Paired-end reads were mapped to the human genome assembly hg19 with TopHat and further analyzed by the Cufflinks-Cuffdiff pipeline to identify differentially expressed transcripts. We ran the pipeline without novel transcript discovery.

Raw data (FASTQ sequences) have been submitted to the National Center for Biotechnology Information (NCBI) Gene Expression Omnibus (GEO) database under the project GSE79820 (submitter M. Pallocca).

### Statistical analysis

Statistical analyzes were performed by using the Student two-tailed t-test to compare *in vitro* experiments. All statistical tests were carried out using GraphPad Prism version 5.0 for Windows, Graphpad Software, San Diego California USA (www.graphpad.com). Probability value of <0,05 was considered statistically significant.

## SUPPLEMENTARY FIGURE AND TABLE


