# Supplementary material for: Deptor transcriptionally regulates endoplasmic reticulum homeostasis in multiple myeloma cells
Source: Oncotarget. 2016 Sep 16;7(43):70546–58. doi: 10.18632/oncotarget.12060 (PMC5342573; doi:10.18632/oncotarget.12060)
Supplement: Supplementary file 1 [file oncotarget-07-70546-s001.pdf]

Deptor transcriptionally regulates endoplasmic reticulum homeostasis in multiple myeloma cells

SUPPLEMENTARY FIGURE AND TABLE

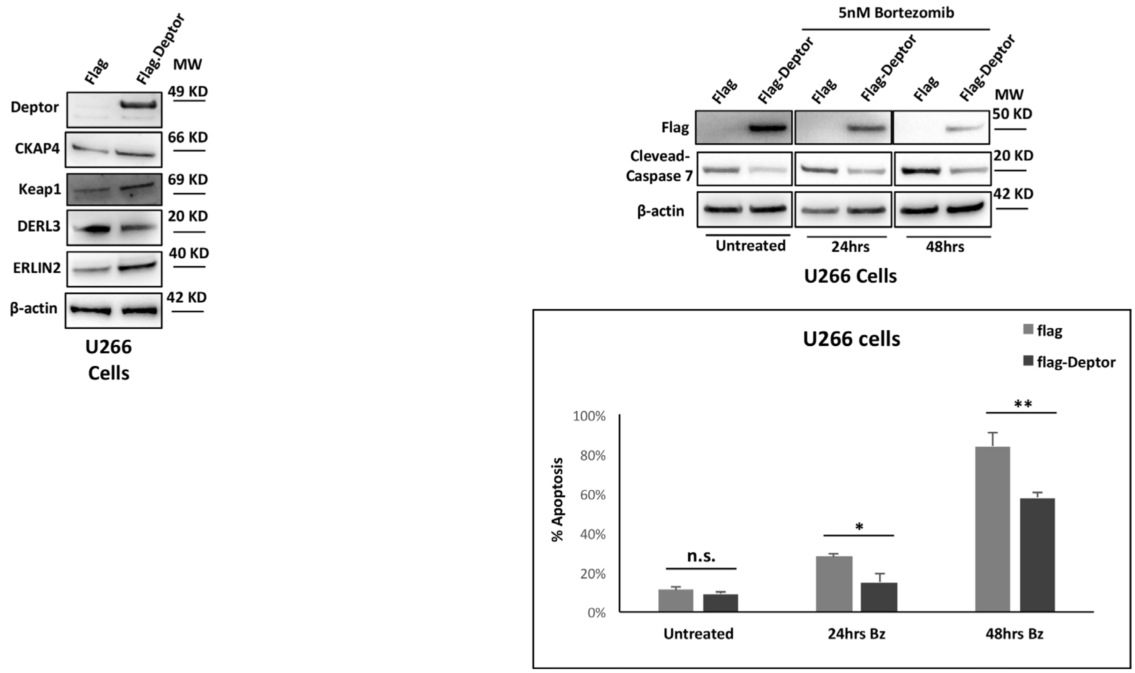

Supplementary Figure S1: Deptor modulates transcription of genes involved in ER homeostasis. A. WB analysis with the indicated Abs of TCEs from U266 cells transiently transfected with Flag-Deptor or empty expression vector. **B.** Top: WB analysis with the indicated Abs of TCEs from U266 cells transfected with as in A and after 24 hrs treated or not with 5nM bortezomib (Bz) for further 24 and 48hrs. Bottom: Cell death detection of U266 cells treated like in B and assayed by trypan blue staining. Percentages represent trypan blue-incorporating cells. Data are presented as the mean SD from three independent experiments performed in duplicate. *P<0.05, **P=0.04.

Supplementary Table S1: Deptor modulates transcription of genes involved in ER homeostasis

Set size	Hits	Pvalue	Goid-Desc	Genes
169	63	0.0015	1: response to endoplasmic reticulum stress (5:5)	BCL2L11, COL4A3BP, CTDSP2, PDIA6, CREB3, CEBPB, YIF1A, SEC61B, SERINC3, COPS5, SEC63, FAM129A, ADD1, TPP1, LRRK2, CREB3L4, CTH, DAB2IP, AARS, DCTN1, DDIT3, SCAMP5, EP300, ERN1, SEC31A, ATF6, UFL1, SDF2L1, GFPT1, IFNG, ACADVL, JUN, LMNA, MAP3K5, NFE2L2, ATP2A2, DERL2, MBTPS2, PML, PARP16, FKBP14, WIPI1, PPP2CB, RNF121, VIMP, MYDGF, TMX4, TRIB3, BCL2, SHC1, CREB3L2, SSR1, TMBIM6, VCP, XBP1, DERL1, TMX1, CASP4, PPP1R15B, DERL3, PDIA4, EDEM1, TATDN2
71	29	0.0078	3: endoplasmic reticulum-Golgi intermediate compartment (5:8)	PDIA6, YIF1A, KDELR1, TMED2, TMED10, TMED1, CHP1, AZIN2, WHAMM, DICER1, TMED3, LAMP5, GJB2, CNIH4, LMAN1, MAN1A1, NUCB2, TMED5, TMED9, STX17, UGGT2, MYDGF, UGGT1, SCYL1, RAB2A, GNPNAT1, SURF4, RAB1B, MCFD2
32	14	0.04	3: endoplasmic reticulum-Golgi intermediate compartment membrane (4:9)	KDELR1, TMED2, TMED10, TMED1, AZIN2, WHAMM, TMED3, LAMP5, LMAN1, TMED5, TMED9, STX17, RAB2A, SURF4
1281	367	0.05	3: endoplasmic reticulum (4:8)	over 100 entries (1, 170, 196,)
91	44	0.0000029	1: establishment of protein localization to endoplasmic reticulum (6:8)	SEC61B, RPL35, SEC63, FAU, SEC11A, MACF1, RPL36, SPCS3, RPL3, RPL12, RPL15, RPL18, RPL22, RPL24, RPL27, RPL29, RPL31, RPL34, RPL35A, RPL37, RPL38, RPL36A, RPLP1, RPN1, RPS3, RPS5, RPS6, RPS9, RPS10, RPS15, RPS17, RPS19, RPS21, RPS25, RPS28, SRP9, SRP14, SRP54, SRP72, SSR1, SSR2, UBA52, RPL14, SEC11C
110	48	0.000053	1: protein localization to endoplasmic reticulum (7:7)	KDELR1, SEC61B, RPL35, SEC63, FAU, SEC11A, MACF1, RPL36, SPCS3, RPL3, RPL12, RPL15, RPL18, RPL22, RPL24, RPL27, RPL29, RPL31, RPL34, RPL35A, RPL37, RPL38, RPL36A, RPLP1, RPN1, RPS3, RPS5, RPS6, RPS9, RPS10, RPS15, RPS17, RPS19, RPS21, RPS25, RPS28, SRP9, SRP14, SRP54, SRP72, SSR1, SSR2, UBA52, GPAA1, RPL14, SEC11C, VAPA, PEX16
886	269	0.005	1: chromosome organization (4:5)	over 100 entries (1, 90, 178,)
61	25	0.01	1: positive regulation of chromatin modification (4:10)	CDK9, RUVBL2, ARID5A, JDP2, CTBP1, MTF2, SNW1, RTF1, LPIN1, SIRT1, PRKD2, ARRB1, KMT2A, MYB, NAP1L2, PML, PRKD1, MAPK3, TGFB1, TPR, VEGFA, XBP1, WDR61, OGT, NCOR1
242	94	0.000011	1: transcription initiation from RNA polymerase II promoter (8:12)	MED6, MED16, TRIM28, CDK7, CDK9, NR2C2AP, COX6B1, COX7C, COX8A, CREBBP, PARP1, DACH1, E2F3, E2F5, AHR, ERCC2, AKT1, ESR1, ESR2, ESRRA, ESRRG, SNW1, NCOA6, NEDD4L, HEY2, SFN, GPI, GSR, GTF2A1, GTF2A2, GTF2B, GTF2E2, GTF2F1, GTF2F2, GTF2H1, GTF2H3, JUNB, SMAD3, SMAD4, SMAD7, NOTCH1, RRM2B, PRKAG2, TRIM33, TAF9B, CYCS, POLR2B, POLR2E, POLR2H, POLR2I, POLR2L, MED1, RNF111, PPM1A, PRKAA1, PRKAB2, CAND1, PTEN, RBL1, RORA, RORC, RXRB, SKI, SKIL, SP1, TAF1, TAF2, TAF4, TCF4, TEAD1, TFDP2, TGIF1, NR2C1, TXN, UBA52, UBE2D1, VDR, YWHAB, YWHAZ, ZNF45, SESN2, MED10, KAT2B, MED30, COX7A2L, MED14, COX5A, MED23, MED26, NCOR1, NCOR2, MAML1, MED12, MED13
83	37	0.00024	1: transcription elongation from RNA polymerase II promoter (8:12)	CDK7, CDK9, SUPT16H, LEO1, DAB2, RNF168, ERCC2, NCBP2, ELL2, RTF1, NELFB, ELP4, GTF2A1, GTF2A2, GTF2B, GTF2E2, GTF2F1, GTF2F2, GTF2H1, GTF2H3, TAF9B, POLR2B, POLR2E, POLR2H, POLR2I, POLR2L, EAPP, SUPT5H, TAF1, TAF2, TAF4, TCEA1, CDC73, WDR61, ELL, IKBKAP, CTDP1

Table relative to Gene Onthology enrichment over all the trancripts significantly correlated with DEPTOR (Minus Set: 2033 probes, Plus Set: 2144, pvalue < 0.01) in the Hanamura MM Dataset of R2.

Full Go Analysis Available at http://hgserver1.amc.nl/cgi-bin/r2/main.cgi?&option=gotestme&factor=1469080954_218858_at_DEPTOR.txt&subset=&species=hs
